# Identification of *Drosophila melanogaster* Strain-Specific Sequence Variations in the *Nepl15* Transcript of Oregon-R relative to FlyBase Data

**DOI:** 10.17912/micropub.biology.002117

**Published:** 2026-05-28

**Authors:** Chase Drucker, Surya Jyoti Banerjee

**Affiliations:** 1 Biological Sciences, Texas Tech University, Lubbock, Texas, United States

## Abstract

According to FlyBase, the *Drosophila melanogaster* (fruit fly) *Neprilysin-like 15 (Nepl15)* gene has only one annotated transcript and a 686-amino acid-long protein. However, *Nepl15* transcripts exhibit organ- and sex-specific differential expression, and *Nepl15* knock-out mutation results in sex-dependent contrasting phenotypes. Thus, we investigated whether unreported sex-specific *Nepl15* transcripts exist. Our sequencing results of *Nepl15* cDNA synthesized from the Oregon-R adult male and female RNA samples show a few changes in the codons, which alter the amino acid sequences in our samples. However, the changes do not impact the subcellular localization or overall Nepl15 protein structure as determined by bioinformatics analyses.

**Figure 1. Alignment of FlyBase Nep1, Nepl15, and Oregon-R Nepl15 protein sequences (A), and predicted 3D structures of FlyBase and Oregon-R Nepl15 proteins (B and C1 to C3). Table 1: List of Neprilysins and the corresponding number of transcripts and unique polypeptides they produce. Table 2: Nucleotide and amino acid sequencing alterations in Oregon-R Nepl15 CDS and protein compared to the corresponding FlyBase data:** **A.** Multiple protein sequence alignment by ClustalW. FBNep1 (the Drosophila melanogaster typical Neprilysin1 protein sequence from FlyBase), FBNepl15 (FlyBase Nepl15 protein sequence), ORNepl15F or ORNepl15M (newly sequenced Oregon-R Nepl15 protein sequences from Female or Male adult flies). Multiple protein sequence alignment of Nep1 (typical Nep in fruit fly), FlyBase (FB) Nepl15, and Oregon-R Nepl15 protein sequences show FBNepl15 and Oregon-R Nepl15 proteins lack the (684) HEXXH and (746) EXXH catalytic motifs that are present in Neprilysin1 (Nep1). Additionally, 5 amino acids are different in the Oregon-R Nepl15 protein (at 15, 18, 199, 239, and 567 positions) from the FB Nepl15 protein sequence (see Table 2). Colored boxes indicate altered amino acid residues in Oregon-R Nepl15 protein relative to FB Nepl15 protein: (blue) indicating the original residue, (green) designating no change at the amino acid residue even though nucleotide sequence was altered, and (red) representing altered amino acid residues. The numbers underneath each colored residue correspond to the amino acid position on the Nepl15 protein sequence. **B.** Predicted 3D protein structure of FB Nepl15 protein made by AlphaFold 3. The box plot shows a predicted aligned errors (PAE) plot, mapping the expected positional error (Å) between pairs of residues, generated by AlphaFold 3. 3D model residues and PAE plot are colored and indicated in the provided key, in which lower values (blue) indicate higher levels of confidence. **C _1_ , C _2_ , and C _3_ .** Overlay of the 3D models of FBNepl15 (blue) and Oregon-R Nepl15 (purple) proteins from three different angles, as generated by AlphaFold 3. Three images display different angles of these two overlapping proteins, without indicating any major change in the Oregon-R Nepl15 protein structure despite the 5 amino acid changes.

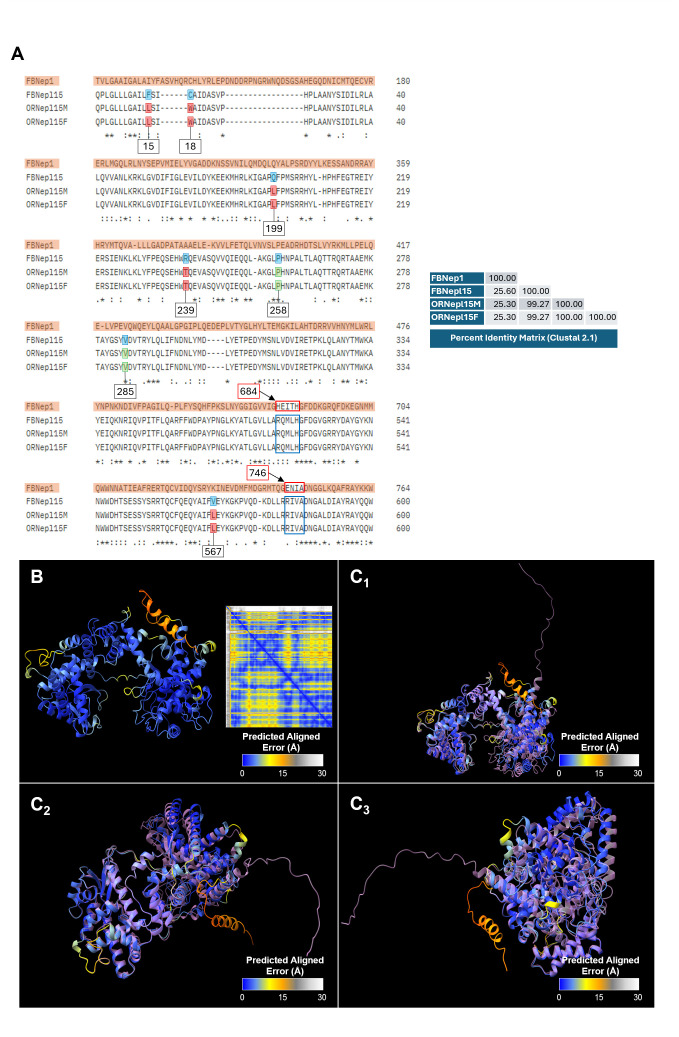



**Table:** 

Table 1. Symbol	Name	Number of Transcripts	Number of Polypeptides
Nep1	Neprilysin 1	4	4
Nep2	Neprilysin 2	3	3
Nep3	Neprilysin 3	3	3
Nep4	Neprilysin 4	3	3
Nep5	Neprilysin 5	4	4
Nep6	Neprilysin 6	2	2
Nep7	Neprilysin 7	1	1
frma	fra mauro	2	2
goe	gone early	3	3
Nepl3	Neprilysin-like 3	1	1
Nepl4	Neprilysin-like 4	1	1
Nepl5	Neprilysin-like 5	1	1
Nepl6	Neprilysin-like 6	1	1
Nepl7	Neprilysin-like 7	2	2
Nepl8	Neprilysin-like 8	1	1
Nepl9	Neprilysin-like 9	1	1
Nepl10	Neprilysin-like 10	1	1
Nepl11	Neprilysin-like 11	2	2
Nepl12	Neprilysin-like 12	1	1
Nepl13	Neprilysin-like 13	1	1
Nepl14	Neprilysin-like 14	1	1
Nepl15	Neprilysin-like 15	1	1
Nepl16	Neprilysin-like 16	1	1
Nepl17	Neprilysin-like 17	1	1
Nepl18	Neprilysin-like 18	1	1
Nepl19	Neprilysin-like 19	1	1
Nepl20	Neprilysin-like 20	1	1
Nepl21	Neprilysin-like 21	1	1
			
<b>Table 2. Genomic Position</b>	<b>Nucleotide Change</b>	<b>Amino Acid Variant</b>	<b>Character Alterations</b>
3R:22766060	T to C	F15L	None
3R:22766049	C to G	C18W	polar - hydrophobic; aromatic
3R:22765507	A to T	Q199L	negatively charged - hydrophobic
3R:22765387	G to C	R239T	Positively charged - polar
3R:22764404	G to T	V567L	None

## Description

Neprilysins (neutral endopeptidases; Neps) belong to the M13 family of Zinc (II) dependent metallopeptidases and were first discovered in mammals (Turner et al., 2001). Typical mammalian Neps, including human Neprilysin (HsNep) encoded by the MME (Membrane Metalloendopeptidase) gene, have a short cytoplasmic N-terminal domain, a shorter hydrophobic transmembrane domain, and a long extracellular C-terminal domain that houses highly conserved motifs. Two of these motifs, HEXXH and EXXAD, are responsible for Zn (II) binding and the subsequent proteolytic cleavage of substrate peptides, typically occurring at the N-terminal side of a hydrophobic residue with a strong preference for Phe or Leu toward the C-terminus (Nalivaeva & Turner, 2013; Sitnik et al., 2014; Turner et al., 2000).

Typical mammalian Neps are normally expressed in a variety of cell types, including brush border cells of the kidneys, brain, neutrophils, lungs, synapses, along with adipocytes and smooth muscle cells present throughout gut tissue, to name a few. Neprilysin cleaves an array of peptides and controls cellular signals that impact the nervous, cardiovascular, inflammatory, immune systems, and nutrient homeostasis (Schling & Schäfer, 2002; Turner et al., 2001).

Phylogenetic studies have identified the presence of Nep orthologs in invertebrate organisms, including *Drosophila melanogaster* (Bland et al., 2008; Sitnik et al., 2014). *Drosophila melanogaster* contains 28 identified neprilysin and neprilysin-like genes (Meyer et al., 2021). Among them, Neprilysin-like 15 (Nepl15), a predicted secreted, catalytically inactive Nep, regulates glycogen and lipid storage in a sex-dependent manner. Previous work with *Nepl15 ^ko^* (knock-out) flies revealed that glycogen and triacylglyceride storage were significantly reduced in adult male mutant flies, while significantly more glycogen storage was observed in adult female mutants, albeit the mutants consume the same amount of food as the controls (Banerjee et al., 2021). Interestingly, a previous study (Banerjee et al., 2021) and transcriptomics data from FlyAtlas2 (Leader et al., 2018) showed that the *Nepl15* transcript is differentially expressed in both larval and adult fly organs, with higher expression in larval fat body and adult head, heart, and fat body. Overall, FlyAtlas shows that the whole adult male body expresses 4.2 times more *Nepl15* than the whole adult female body. Additionally, overexpression of *Nepl15* in the fat body increased triglyceride levels only, whereas its overexpression in the gut increased glycogen levels only in adult male flies, suggesting *Nepl15* has tissue-specific variable effects (Banerjee et al., 2021). Interestingly, the fly database (FlyBase.org) reports that the *Nepl15* gene in *D. melanogaster* ISO1 MT (dm6) ( *y ^1^ ; cn ^1^ bw ^1^ sp ^1^* ) strain has no intron and produces only one transcript. However, 10 other fly *Neprilysin* and *Neprilysin-like* genes encode more than one transcript (Table 1) (FlyBase.org).

Therefore, we asked the following questions: Are there any unreported *Nepl15* transcript variants present in wild-type (Oregon-R) adult male and female flies that produce different Nepl15 protein isoforms in the two sexes? Are there any differences in the 5′ and 3′ untranslated regions (UTRs) in the *Nepl15* transcript which may contribute to its post-transcriptional regulation? To answer these questions, we isolated total RNA from the whole-body of adult male and virgin female Oregon-R flies (FBsn0000276) and synthesized cDNA. Next, we took PCR based approach to amplify the *Nepl15* CDS (coding sequence, 2061 bps) and its transcript (2213 bps including the 5′ and 3′ UTRs), followed by sequencing them and aligning the sequences with the corresponding sequences in FlyBase.

We did not find any nucleotide sequence difference at the 5′ and 3′ UTRs of the Oregon-R and FlyBase Nepl15 transcript sequences. However, sequencing results show that the Oregon-R *Nepl15* CDS from male and female flies contains the exact 7-nucleotide sequence changes compared to the FlyBase sequence. These changes translate into only 5 amino acid changes, among which 3 changes include amino acids with different properties (Table 2) ( Figure 1A ). The Oregon-R adult male and female Nepl15 (ORNepl15M and ORNepl15F) protein sequences were aligned with the FlyBase Neprilysin1 (FBNep1) and Nepl15 (FBNepl15) protein sequences using ClustalW to show the changed 5 amino acid residues (Table 2) ( Figure 1A ). Further bioinformatics analysis of the Oregon-R Nepl15 protein reveals that the amino acid changes did not impact the Nepl15 predicted cleavage site (between amino acids 22 and 23) (SignalIP and DeepTMHMM) (Hallgren et al., 2022; Teufel et al., 2022), and extracellular localization (DeepLoc) (Ødum et al., 2024). Additional analysis of 3D models of the FlyBase Nepl15 ( Figure 1B, blue ribbon diagram) and Oregon-R Nepl15 proteins utilizing AlphaFold 3 (Abramson et al., 2024) shows no significant change in their structures in their overlays (blue and purple ribbons depict the Nepl15 3D protein models in FlyBase strain and Oregon-R, respectively) (Figures 1 C _1_ – C _3_ ).

Collectively, these results reveal that a slightly different amino acid sequence in the Nepl15 protein is present in our lab-grown wild-type Oregon-R flies as compared to the FlyBase Nepl15 protein, indicative of a strain-specific amino acid sequence variation in the same protein. Bioinformatic analyses do not indicate any major structural or functional shifts in the Oregon-R Nepl15 protein due to these changes. Thus, our study confirms the presence of only one *Nepl15* transcript version in *D. melanogaster* Oregon-R adult male and female flies. Although, the changes in the nucleotide sequences within the Oregon-R *Nepl15* codons and corresponding amino acid changes in the Nepl15 protein may not impact the protein's localization, structure, and function, still our research outcome can contribute to future investigations in two significant ways: (i) this report will help to perform primer design, PCR, qPCR, gene cloning, guide DNA design for CRISPR, and site specific mutation accurately for Nepl15 gene in Oregon-R fly strain; and (ii) if researchers use a *Drosophila* species or strain other than the one used in a database or previously published work, our result recommends that it is important to verify the DNA sequence before designing the components of molecular biology based experiments.

## Methods

***Drosophila melanogaster* husbandry**

*Drosophila* *melanogaster* Oregon-R strain was maintained on Bloomington's fly food formulation (Genesee Scientific Nutri-Fly_66-116) at 25 °C. 5 to 7 days old male and virgin female adult flies were used for RNA extraction.

**FlyBase Data:**

The FlyBase website, http://flybase.org, was used to retrieve the Nepl15 transcript and CDS sequences. The genome release number is FB2026_01 (March 12, 2026).

**RNA isolation and cDNA synthesis**

Total RNA was extracted from 5 to 7 days old, 25 male and 25 virgin female Oregon-R adult flies using TRI reagent (Sigma T9424) (Banerjee et al., 2021). The RNA sample purity was validated by measuring 260/280 and 230/280 ratios, and the RNA integrity was tested by gel electrophoresis. The cDNA synthesis was then performed using 5x iScript Reaction Mix (Bio-Rad 1708889) following the manufacturer's protocol.

**PCR and Purification**

Specific forward (FP) and reverse primer (RP) pairs (Table 3) were designed using the Serial Cloner (v2.6.1) (Branco & Choupina, 2021) to amplify *Nepl15* CDS and transcript using the OR male and female cDNA templates separately. The primer sequences were designed based on the FlyBase *Nepl15* CDS and Transcript sequences. PCR was performed using Q5 High-Fidelity 2X Master Mix (New England Biolabs M0492S) following the manufacturer's protocol. The PCR products were purified using the NucleoSpin Gel and PCR clean-up kit (Macherey-Nagel 740609) following the manufacturer's instructions.

Table 3. List of primers used for PCR.

**Table:** 

**Primer**	**Sequence (5' to 3')**
Nepl15_CDS_FP	ATGGCCTCTCAACCGCTTG
Nepl15_CDS_RP	TCAGTAGAGAGTGCACTTAAAGCG
Nepl15_T.SCRPT_FP	CCCGCTCAGTTTCGTTTC
Nepl15_T.SCRPT_RP	ATGACAAGTGCAACAAATGCTTTT

**DNA Sequencing Analysis**

We designed several primers (listed in Table 4) to cover the entire length of the *Nepl15* CDS or transcript. Sanger sequencing of the purified PCR samples was performed by Azenta Life Sciences (genewiz.com). The high-quality sequence contigs were aligned to the corresponding FlyBase sequence using SerialCloner (v2.6.1) (Branco & Choupina, 2021).

Table 4. List of primers used for sequencing.

**Table:** 

**Primer**	**Sequence (5' to 3')**
Nepl15_CDS_FP1	TGAAACGCAAGCTTGGCGTG
Nepl15_CDS_FP2	GCTTTGGAAGCGTTGGATAT
Nepl15_CDS_FP3	TGGAATACAAGGGTAAGCCG
Nepl15_CDS_RP1	TATTCAGGGCGGATCGCGTG
Nepl15_T.SCRPT_FP1	GGTTGGCCGGAGATTCGTGT
Nepl15_T.SCRPT_FP2	CATGTCAAATCTGGTGGATG
Nepl15_T.SCRPT_FP3	TGGGATCACACCTCGGAGAG
Nepl15_T.SCRPT_RP1	GGCACTGTTCTCAGCTCCTC

**Protein sequence analysis and other bioinformatics analysis**

Changes in the Oregon-R *Nepl15* CDS sequences compared to the FlyBase sequence were identified using the Serial Cloner (v2.6.1) (Branco & Choupina, 2021). Variations in the *Nepl15* codon sequences between Oregon-R CDS and FlyBase CDS sequences were analyzed using Serial Cloner's built-in BLASTn and BLASTp alignment tools. Next, Clustal Omega (Madeira et al., 2024) was used to align the FlyBase Neprilysin 1 (DmNep1, a typical fly neprilysin, orthologous to human neprilysin), Oregon-R male and female Nepl15, and FlyBase Nepl15 protein sequences to identify changes in Oregon-R Nepl15 protein sequences. The newly sequenced Oregon-R Nepl15 protein sequence was analyzed for any change in its subcellular localization, signal sequence site, and 3D structure compared to those of the FlyBase-reported Nepl15 protein sequence using different bioinformatics tools like DeepLoc2.1 (Ødum et al., 2024), SignalP – 6.0 (Teufel et al., 2022), and DeepTMHMM – 1.0 (Hallgren et al., 2022), respectively.

**AlphaFold 3 Protein Prediction**

Protein structure predictions for Nepl15 were generated using AlphaFold 3 within the ChimeraX interface. The amino acid sequence of the Oregon-R Nepl15 protein was used as input. Structure prediction was performed in ChimeraX (version 1.11.1) using the following workflow: Tools to Structure Prediction to AlphaFold, after which the protein sequence was entered into the “Sequence” field and submitted for prediction. The “don't minimize” parameter was selected before the sequence in the Google Collab window to prevent minimization during model generation, consistent with previously described protocols. Multiple models were generated, and model confidence was evaluated using predicted aligned error (PAE) metrics. The structure with the highest overall confidence, defined by the lowest PAE values and highest predicted local distance difference test scores, was automatically selected for further visualization and structural alignment analyses (Abramson et al., 2024; Meng et al., 2023).

## Reagents

All reagents' names, with the company names and catalog numbers, are mentioned in the methods.
